# The Influence of Genetic Drift and Selection on Quantitative Traits in a Plant Pathogenic Fungus

**DOI:** 10.1371/journal.pone.0112523

**Published:** 2014-11-10

**Authors:** Tryggvi S. Stefansson, Bruce A. McDonald, Yvonne Willi

**Affiliations:** 1 Plant Pathology, Institute of Integrative Biology, ETH Zürich, Zürich, Switzerland; 2 Evolutionary Botany, Institute of Biology, University of Neuchâtel, Neuchâtel, Switzerland; University of Idaho, United States of America

## Abstract

Genetic drift and selection are ubiquitous evolutionary forces acting to shape genetic variation in populations. While their relative importance has been well studied in plants and animals, less is known about their relative importance in fungal pathogens. Because agro-ecosystems are more homogeneous environments than natural ecosystems, stabilizing selection may play a stronger role than genetic drift or diversifying selection in shaping genetic variation among populations of fungal pathogens in agro-ecosystems. We tested this hypothesis by conducting a *Q*_ST_/*F*_ST_ analysis using agricultural populations of the barley pathogen *Rhynchosporium commune.* Population divergence for eight quantitative traits (*Q*_ST_) was compared with divergence at eight neutral microsatellite loci (*F*_ST_) for 126 pathogen strains originating from nine globally distributed field populations to infer the effects of genetic drift and types of selection acting on each trait. Our analyses indicated that five of the eight traits had *Q*_ST_ values significantly lower than *F*_ST_, consistent with stabilizing selection, whereas one trait, growth under heat stress (22°C), showed evidence of diversifying selection and local adaptation (*Q*_ST_>*F*_ST_). Estimates of heritability were high for all traits (means ranging between 0.55–0.84), and average heritability across traits was negatively correlated with microsatellite gene diversity. Some trait pairs were genetically correlated and there was significant evidence for a trade-off between spore size and spore number, and between melanization and growth under benign temperature. Our findings indicate that many ecologically and agriculturally important traits are under stabilizing selection in *R. commune* and that high within-population genetic variation is maintained for these traits.

## Introduction

Quantitative genetic variation within and among populations is affected by the evolutionary processes of mutation, migration, genetic drift and selection [1]. Empirical studies so far support the ubiquitous role of genetic drift and diversifying selection for shaping population genetic structure within species [2]–[4]. However, organisms living in human-altered environments, particularly those in agro-ecosystems, may experience a difference in the relative impact of these evolutionary forces. On the one hand, large habitat sizes may reduce the importance of genetic drift. On the other hand, the selection regime may be similar among geographically distant populations, leading to more stabilizing selection. We tested these predictions by inferring the roles of genetic drift and type of selection in the fungal barley pathogen *Rhynchosporium commune*. We also estimated within-population genetic variation in quantitative traits and at neutral marker loci to assess the pathogen’s potential to adapt. The ability of a fungal plant pathogen to adapt to the deployment of resistant hosts, to fungicides and to changes in climate are considered to be important contributors to a pathogen’s overall risk of causing extensive damage in agro-ecosystems [5].

Global anthropogenic homogenization of environmental conditions has recently been recognized as important for the emergence of new diseases and the spread of invasive species [6]–[9]. In some cases, the success of such species may be based on a sampling effect – at some point the species comes into contact with the new, human-altered habitat and spreads. But in many cases, the success is probably linked to adaptation to a habitat that humans created in abundance by homogenizing of environmental conditions. An example is globally distributed agro-ecosystems that have favoured the emergence and spread of diseases [7]. The relative importance of individual evolutionary processes is likely to be systematically different between organisms that occupy such homogeneous agro-ecosystems and organisms that occupy more heterogeneous natural habitats. First, large habitat size is likely to increase effective population size. As population size is inversely related with genetic drift [10], populations of such species should be less exposed to genetic drift in the long run. Second, the homogenization of environmental conditions should streamline selection regimes across populations, leading to stabilizing selection among them. Finally, the combination of large population sizes, more cycles of reproduction per year and similar selection processes operating across populations may generally increase within-population genetic variation and evolutionary potential.

Genetic drift and selection are predicted to have different impacts on genetic variation within and among populations, and this can be used to infer their relative importance [11]. Specifically, the comparison of population genetic divergence for neutral marker loci (*F*_ST_) with that for quantitative traits (*Q*_ST_; the ratio of among-population genetic variation to the sum of among- and within-population genetic variation) reveals the predominant evolutionary force. This approach is based on the prediction that the difference between the two measures of divergence is due to selection acting on the quantitative traits. Higher *Q*_ST_ values than *F*_ST_ values are interpreted as diversifying selection for local adaptation. Lower *Q*_ST_ values than *F*_ST_ values are interpreted as stabilizing selection among populations, potentially associated with directional selection operating in the same way in each population. A correlation between the two measures indicates the signature of genetic drift.

Several meta-analyses have been performed on *Q*_ST_/*F*_ST_ studies [2]–[4], [12]. One of the most recent ones shows that across studies, divergence in expressed traits and in neutral markers is generally positively correlated, implying that these traits are affected by genetic drift [4]. The other general finding is that *Q*_ST_ is often larger than *F*_ST_, suggesting that diversifying selection among populations is the common type of selection acting among populations. This meta-analysis also clearly documents a strong bias in study organisms towards wild species of plants and animals and a deficiency of studies using fungi and pathogens in anthropogenically-altered environments [4], which may in part explain the finding of the predominance of genetic drift and diversifying selection.

The goal of this study was to elucidate the relative roles of genetic drift and selection on population genetic divergence and to link genetic variation within populations at neutral marker loci with that for quantitative traits. Results are discussed in the context of the evolutionary forces shaping within- and among-population genetic variation in an agricultural fungal pathogen. Comparisons between populations were made within a *Q*_ST_/*F*_ST_ context and measures of within-population genetic variation were estimated for several traits that are thought to play a role in the amount of damage caused by the pathogen. Experiments were performed using nine field populations of the barley pathogenic fungus *Rhynchosporium commune* collected from nine countries. The traits included a classic morphological trait – spore size, fitness-related life-history traits – spore number and virulence *in planta*, physiological traits – *in vitro* thermal performance under three temperatures, and a trait related to agricultural practices – fungicide resistance.

## Materials and Methods

### The species and populations studied

*Rhynchosporium commune* is the causal agent of a serious disease called barley scald, which is found in all major barley-growing areas of the world. Barley scald causes severe yield loss and economical damage [13], [14]. The pathogen is haploid and molecular evidence indicates that it undergoes sexual reproduction although a teleomorph has never been reported [15]. *Rhynchosporium commune* specifically infects *Hordeum* species, including domesticated and wild barley as well as *Bromus diandrus* [16]. The species is thought to have emerged in northern Europe around 3000 years ago following the introduction of barley agriculture and to have spread outside of Europe only recently, likely through anthropogenic seed dispersal [15], [17].

Nine populations from naturally infected barley fields in nine countries were chosen from a global collection of *R. commune* and are described in previous publications [18], [19]. The nine countries were New Zealand (NZ), Australia (AU), Ethiopia (ET), Spain (SP), Switzerland (CH), Finland (FI), Norway (NO), Iceland (IS) and USA (US). The populations came from locations that differed for local climatic conditions, agricultural practices and length of the host growing season. Fourteen randomly chosen, genetically distinct isolates per population were used in this study with three clonal replicates of each isolate per treatment. For each experiment the isolates were revived from −80°C storage by plating on Lima Bean Agar (LBA, 60 g/L lima beans, 12 g/L agar, 50 mg/L kanamycin). To comply with a common-garden experimental design all isolates were handled on the same day by the same people for each of the experimental steps described below. To minimize systematic differences in the treatment of isolates, the growth medium was prepared as a single batch that was poured into Petri plates on the same day, the Petri plates were randomized before inoculation, and isolates from different populations were randomized before inoculation onto the plates.

### *In vitro* assessment of traits

The following traits were assessed *in vitro*: growth under different temperatures and fungicide concentrations, colony melanization, spore size and spore number. For all assessments, isolates were first grown for 10–14 days at 18°C in darkness and then transferred to fresh LBA plates, where growth occurred for another two weeks. Spores were harvested by adding 1.5 ml of sterile water and scraping, and then counted using a haemocytometer.

Methods used to assess growth rate at different temperatures were described earlier [18]. Briefly, 100 µl of spore solution (1000 spores/ml) were placed on nine standardized 60 mm Potato Dextrose Agar plates (PDA, 24 g/L potato dextrose, 15 g/L agar, 50 mg/L kanamycin). Spores were spread with a sterile glass rod. Following inoculation, plates were left to dry for 15–20 minutes and then three plates per isolate were incubated in darkness at 12°C, another three at 18°C, and a further three at 22°C. Fungal colonies on each plate were photographed with a digital camera after 12, 15, 18, 21, 24, 34 and 44 days of growth under standardized conditions and the colony size (mm^2^) was measured with the image analysis software APS Assess [20]. Preliminary analyses of the growth trajectory revealed that growth followed a power function between day 12 and day 24 across all isolates and populations. Therefore, growth rate was calculated for each individual replicate as the slope of the regression of log-transformed colony size against log-transformed time, between days 12 and 24 (PROC REG in SAS [21]).

For the assessment of fungicide resistance, 100 µl of a 1000 spores/ml spore solution were spread on plates amended with fungicide (0.1 ppm in one experiment and 0.025 ppm cyproconazole in a second experiment) and without fungicide (in both experiments). Each isolate-treatment combination was replicated three times and plates were incubated at 18°C. Growth rates were measured using digital images as described above. Fungicide resistance was measured by dividing the colony growth rate in the presence of fungicide by the colony growth rate in the absence of fungicide. This calculation was performed by pairing up all possible replicate combinations of the two treatments (with/without fungicide), creating 9 pseudo-estimates per isolate. Because fewer than 20% of the isolates showed detectable growth at the higher fungicide concentration, data were analysed using the average relative growth rate of randomly paired replicates of the two fungicide experiments for an isolate, totalling a maximum of 9 estimates per isolate. When in one of the treatments no growth was observed, a value of 0 for that treatment was assumed.

Colony melanization on each plate used in the growth rate experiment (12°C, 18°C, 22°C) and the fungicide resistance experiment with 0.025 ppm cyproconazole (without control plates) was measured using Adobe Photoshop vCS5 (Adobe Systems) as the mean grey value of the colonies after 34 days of growth. Because grey values were significantly and positively correlated across all four environments, we calculated melanization for each replicate as the mean across all environments.

For the assessment of spore size and spore number, mycelium was transferred to three standardized LBA plates per isolate. Because isolates from Spain and Iceland did not produce enough mycelia to be transferred to new plates, they were excluded from this experiment. Mycelium was spread using a sterile metal loop to cover the surface of each plate. After 14 days of growth at 18°C in darkness, four 49 mm^2^ agar plugs were removed from each plate and placed in 8 ml plastic tubes containing 5 ml of 1% sodium dodecyl sulfate (SDS) solution. Tubes were vortexed for 30 seconds to release spores from the agar surface and 20 µl was pipetted into a disposable Cellometer counting chamber (Nexcelcom Bioscience, type SD100). Spore number (spores/ml) and mean spore size (µm) were calculated automatically using the Cellometer Auto T4 cell counter (Nexcelcom Bioscience).

### *In planta* assessment of virulence

Methods used to assess virulence *in planta* were described earlier [19]. Briefly, the spore concentration of each isolate was standardized to 10^6^ spores/ml and adjusted to a total volume of 5 ml. Virulence was measured on the spring barley cultivar Beatrix (Viskosa×Pasadena), which was rated as moderately susceptible to *R. commune*. For each isolate, three pots were sown with four seeds in each pot in a single greenhouse chamber (14 h light/18°C, 8 h dark/15°C, constant relative humidity of 60%). After 12 days of growth the second leaf was fully emerged. Plants were then inoculated in a sterile chamber with 5 ml of spore suspension per isolate. They were allowed to dry for 15–20 minutes and arranged randomly within mobile humidity chambers that were placed within the growth chamber. Using ultrasonic humidifiers the relative humidity was kept at 100% for 48 hours. After the humidity chambers were removed, the pots were randomly arranged in the greenhouse chamber and the relative humidity was set to 60%. Fifteen days after inoculation, the second leaf on all plants was cut and fixed to a paper sheet. Digital images were taken, the percentage of leaf area affected by the disease (virulence) was measured using the image analysis software APS Assess [20], and means per pot were calculated. A logit transformation was applied to the data to approximate a normal distribution prior to variance analysis.

### Microsatellite genotyping

A microsatellite analysis was performed on the same 126 isolates as described previously [22] using eight selectively neutral loci: Rh1, Rh2, Rh4, Rh5, Rh6, Rh8*, Rh11 and Rh14 [15]. Alleles were assigned using the software GeneMapper v4.1 (Applied Biosystems). Multi-locus genotype data revealed that all isolates were unique. Genetic variation was estimated using measures of gene diversity [23], allelic richness and number of unique alleles with the program FSTAT v2.9.3 [24]. Population differentiation for the microsatellite loci (*F*_ST_) was assessed both pairwise and overall and standard deviations for overall *F*_ST_ values were calculated by jackknifing over loci using FSTAT v2.9.3 [24].

### Data analysis

A general linear model analysis was performed to determine whether there were significant effects of isolate and population on trait values (PROC GLM in SAS [21]), where population was tested over isolate nested within population. The model was Y = M+P+I(P)+E where Y refers to the trait value of a replicate for isolate I nested within population P, and M was the overall mean. In a common-garden experiment with clonal propagules, variance among replicates of clones (E) can be interpreted as environmental variance because replicates have the same genotype. In contrast, both variance among populations and variance among isolates within population represent genetic variances. Furthermore, given that the species is haploid and dominance is therefore absent, we assumed that genetic variances were additive. If epistasis plays a role, within-population estimates reflect upper limits for additive genetic variance, and *Q*_ST_ values may be somewhat lower than if gene effects were purely additive [25].

Population differentiation for quantitative traits (*Q*_ST_) was compared to population differentiation for neutral microsatellite markers (*F*_ST_) to infer the relative role of selection and neutral evolution in shaping population divergence. Population differentiation was calculated as the ratio of genetic variance among populations to the sum of genetic variances within and among populations using a haploid-modified version of the equation introduced by Spitze [11], [26]. The variance component analysis was done using the model Y = M+P+I(P)+E (PROC MIXED in SAS, method = reml [21]) and standard errors (SE) for *Q*_ST_ values were obtained by jackknifing over populations [27], [28]. Both overall and pairwise *Q*_ST_ values were calculated. For overall *Q*_ST_ and *F*_ST_, 95% confidence intervals (approximated by ± 2 SE) were compared to determine whether differences were significant. For pairwise *Q*_ST_ and *F*_ST_ values, Mantel tests were performed with the program GenAIEx v6.5 [29] to determine whether correlations were significant.

Within-population genetic variation for quantitative traits was estimated by genetic variance and heritability. Variance components were calculated for each trait and population using the model Y = M+I+E (PROC MIXED in SAS, method = reml [21]) and the standard errors were obtained by jackknifing over isolates within each population. Heritability (*h* ^2^) was calculated as the ratio of genetic variance (*V*_G_) to total phenotypic variance within populations. To examine correlations among traits, trait values of each trait-population combination were standardized to a mean of 0 and a standard deviation of 1, and then correlation analyses between traits were performed based on isolate means (PROC CORR in SAS [21]). Furthermore, we tested for correlations between Nei’s gene diversity for the neutral SSR markers and quantitative genetic variation depicted by trait-specific *h* ^2^ and *V*_G_ as well as by their means across traits. The correlation on mean *V*_G_ across traits was based on trait-specific *V*_G_ that had been centered to a mean of 0 and a standard deviation of 1 beforehand. Significance of correlations was based on two-tailed tests.

## Results

### Trait variation

Populations differed significantly for all traits except melanization, spore number and spore size, while isolates nested within populations differed significantly for all eight traits (Table 1). Differences in trait means between populations as indicated by coefficients of variation were highest for virulence, followed by fungicide resistance, growth rate at 22°C and spore number (Table 2).

**Table 1:** General linear model analyses testing the effect of population and isolate (nested within population) on quantitative traits of *Rhynchosporium commune*.

Trait	Source	df	MS	*F*	*P*
Growth rate 12°C	Population	8	0.44	4.35	0.0002
	Isolate	103	0.10	5.50	<0.0001
	Error	189	0.02		
Growth rate 18°C	Population	8	0.37	2.29	0.0267
	Isolate	102	0.16	7.06	<0.0001
	Error	191	0.02		
Growth rate 22°C	Population	8	0.88	7.61	<0.0001
	Isolate	97	0.12	9.58	<0.0001
	Error	168	0.01		
Fungicide resistance	Population	8	2.09	5.48	<0.0001
	Isolate	91	0.38	30.20	<0.0001
	Error	587	0.01		
Melanization	Population	8	1.19	1.10	0.3678
	Isolate	111	1.08	18.10	<0.0001
	Error	225	0.06		
Spore size	Population	6	6.44	1.17	0.3276
	Isolate	87	5.48	5.91	<0.0001
	Error	177	0.93		
Spore number	Population	6	7154.7	1.99	0.0754
	Isolate	87	3591.7	13.69	<0.0001
	Error	187	262.4		
Virulence	Population	8	56.65	2.49	0.0164
	Isolate	105	22.77	17.19	<0.0001
	Error	211	1.32		

Population effects were tested over isolates nested within population and isolate effects over the pooled error. Samples sizes for the eight traits listed were 301, 302, 274, 687, 345, 271, 281, and 325, respectively.

**Table 2:** Nei’s gene diversity based on eight microsatellite markers and least squares means for quantitative traits.

Population	Gene diversity	Growth rate 12°C	Growth rate 18°C	Growth rate 22°C	Fungicide resistance	Melanization	Spore size	Spore number	Virulence
AU	0.69 0.14	0.83 0.29	1.05 0.11	0.61 0.14	0.40 0.23	1.53 0.76	9.69 0.78	66.9 32.6	61.8 37.2
CH	0.49 0.36	1.05 0.29	1.07 0.33	0.90 0.22	0.66 0.43	1.48 0.43	9.12 2.13	70.3 51.4	48.4 34.3
ET	0.58 0.27	0.99 0.23	1.03 0.29	0.33 0.31	0.29 0.25	1.45 0.56	9.61 1.71	39.2 29.2	33.9 37.2
FI	0.80 0.09	1.19 0.15	1.09 0.16	0.96 0.21	0.42 0.21	1.67 0.64	9.33 1.25	57.9 29.9	27.9 32.5
IS	0.61 0.15	0.89 0.20	0.93 0.29	0.61 0.22	0.26 0.30	1.74 0.67	-	-	43.3 36.9
NO	0.78 0.12	1.13 0.16	1.16 0.20	0.82 0.18	0.39 0.24	1.54 0.40	9.44 0.98	54.0 30.0	30.6 34.3
NZ	0.76 0.12	0.88 0.21	0.91 0.31	0.62 0.15	0.83 0.28	1.89 0.89	9.08 0.84	51.3 30.7	12.2 15.0
SP	0.77 0.09	1.01 0.19	1.25 0.16	0.62 0.31	0.51 0.24	1.28 0.47	-	-	51.6 26.6
US	0.64 0.19	1.04 0.15	0.95 0.20	0.61 0.29	0.48 0.12	1.44 0.53	10.29 2.03	34.6 34.2	27.1 29.6
Mean	0.68	1.00	1.05	0.67	0.47	1.56	9.51	53.5	37.4
SD	0.11	0.12	0.11	0.19	0.18	0.18	0.41	13.2	15.2
CV	0.16	0.12	0.11	0.28	0.38	0.12	0.04	0.25	0.41

For each *Rhynchosporium* population (abbreviated by the country’s name) and trait, least squares mean (top line) and standard deviation (calculation based on isolate means; bottom line) are reported. The last three rows contain the mean, standard deviation (SD) and coefficient of variation (CV) across populations for each trait. For virulence, original, non-transformed data was used to calculate population means.

### Variation at neutral marker loci

Microsatellite analysis was performed to assess overall and pairwise genetic differentiation among populations as well as within-population gene diversity at neutral loci. The overall *F*_ST_ value across populations was 0.21±0.07 (standard deviation). Pairwise *F*_ST_ ranged from 0.002 between the two Scandinavian populations of Norway and Finland to 0.42 between Switzerland and Ethiopia (Table S1). Norway and Finland also had the lowest mean pairwise differentiation from all other populations and Switzerland and Ethiopia the highest. Nei’s gene diversity was highest in the populations from Finland (0.80) and Norway (0.78) and lowest in the populations from Switzerland (0.49) and Ethiopia (0.58) (Table 2).

### *Q_ST_-F_ST_* comparisons

**Figure 1:**
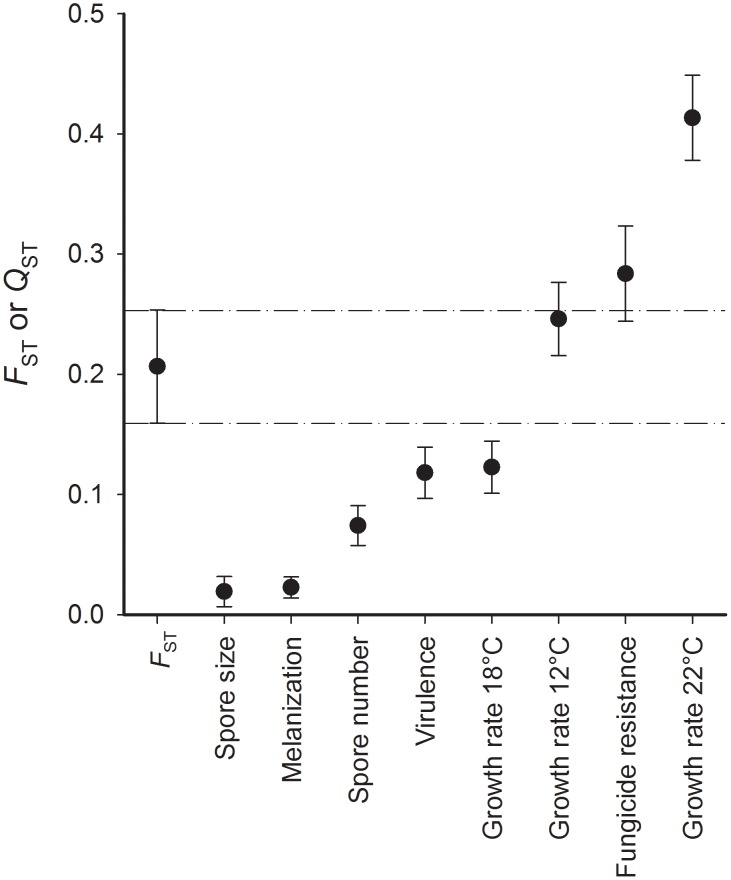
Comparison of genetic divergence estimated by *F*_ST_ and by *Q*_ST_ for eight quantitative traits of populations of *Rhynchosporium commune*. *Q*_ST_ values were statistically different from *F*_ST_ except for growth rate at 12°C and fungicide resistance. Bars represent the 95% confidence interval. The range within the two dashed lines indicates the 95% confidence interval of overall *F*_ST_. Traits with *Q*_ST_ below that range are interpreted as being under stabilizing selection, while traits with *Q*_ST_ above that range are interpreted as being under diversifying selection. When *Q*_ST_ and its confidence interval overlap with the *F*_ST_ range between the dashed lines, neutral evolution acting among populations cannot be excluded.

To evaluate the relative influence of genetic drift and selection in shaping trait variation across populations, we compared *F*_ST_ with population differentiation in quantitative traits (*Q*_ST_). We found that overall *Q*_ST_ was significantly lower than overall *F*_ST_ (Fig. 1) for five of the traits: lowest for spore size, followed by melanization, spore number, virulence and growth rate at 18°C. The two estimates were not significantly different for growth rate at 12°C and fungicide resistance, and *Q*_ST_ was significantly higher than *F*_ST_ for growth rate at 22°C. Mantel tests between pairwise *Q*_ST_ and *F*_ST_ values revealed no significant correlation for six traits (*r*_MANTEL_ for growth rate 22°C: 0.05, fungicide resistance: 0.10, melanization: −0.23, spore size: −0.26, spore number: 0.31, virulence: −0.20). For the remaining two traits, the correlation was significantly negative, for growth rate at 12°C (*r*_MANTEL_ = −0.58, *P* = 0.01) and for growth rate at 18°C (*r*_MANTEL_ = −0.41, *P* = 0.04) (pairwise *Q*_ST_ values in Table S1).

### Heritabilities and trade-offs

**Figure 2:**
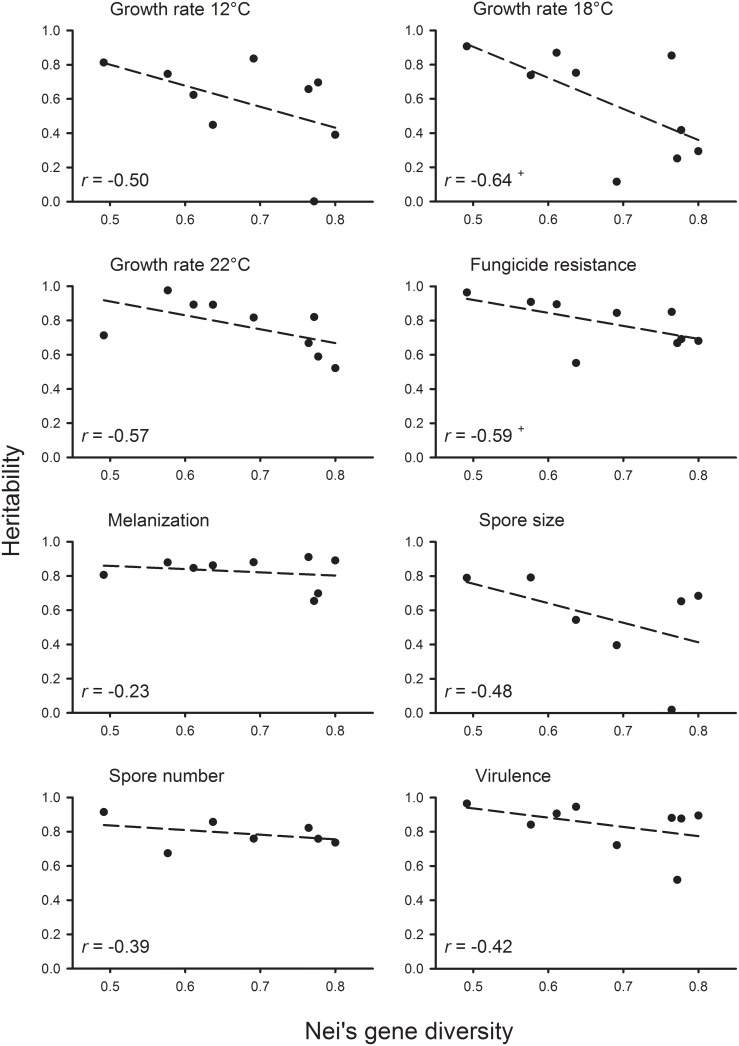
Relationship between Nei’s gene diversity and heritability based on nine (or seven) populations of *Rhynchosporium commune* for the eight traits studied. Pearson correlation coefficients, *r*, were non-significant for individual traits (^+^ *P*<0.1) but highly significant for average heritability across traits (*r* = −0.82, *P*<0.01).

**Figure 3:**
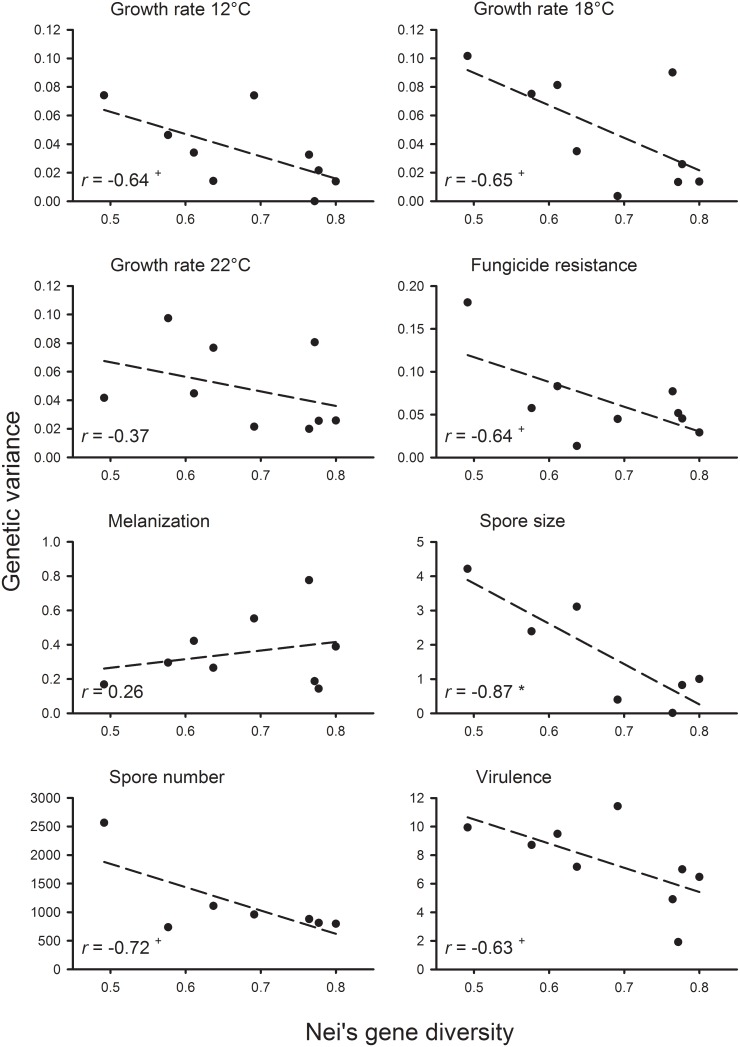
Relationship between Nei’s gene diversity and genetic variance based on nine (or seven) populations of *Rhynchosporium commune* for the eight traits studied. Pearson correlation coefficients, *r*, were non-significant for most individual traits (^+^ *P*<0.1, *P<0.05) but highly significant for average genetic variance across traits (*r* = −0.92, *P*<0.001). Average genetic variance was calculated by first standardizing genetic variances for each trait to a mean of 0 and a standard deviation of 1.

Estimates of heritability were generally high across all eight traits (Table 3). The highest mean heritability values were found for virulence (0.84) and melanization (0.82) and the lowest for spore size (0.55), growth rate at 12°C (0.58), and growth rate at 18°C (0.58). The three traits that showed the lowest mean heritability also had the highest variation between population heritability values, as measured by the coefficient of variation. The lowest coefficients of variation for heritability among populations were found for spore number and melanization. The population from Switzerland had the highest heritability for four out of the eight traits measured and the highest average heritability across traits. The population from Spain had the lowest heritability for three out of six traits and the lowest average heritability. The correlation analysis among isolate means for each trait revealed similar numbers of positive and negative correlations among traits (Table 4). Three of them were significant after Bonferroni-correction of the α-value. Growth rates at 18°C and 12°C were positively correlated while the trait pairs of spore size and spore number, and growth rate at 18°C and melanization were negatively correlated. Although not statistically significant for any individual trait, correlations between gene diversity and heritability were all negative (Fig. 2). Similarly, although only significant for spore size, correlations between gene diversity and genetic variance were generally negative (Fig. 3). When genetic variation estimates were averaged across traits, the association was significantly negative for both heritability (*r* = −0.82, *P*<0.01) and genetic variance (*r* = −0.92, *P*<0.001).

**Table 3:** Heritability estimates and standard deviations from jackknifing over isolates for the eight quantitative traits.

Population	Growth rate 12°C	Growth rate 18°C	Growth rate 22°C	Fungicide resistance	Melanization	Spore size	Spore number	Virulence
AU	0.84 0.02	0.12 0.08	0.82 0.06	0.84 0.05	0.88 0.04	0.40 0.02	0.76 0.03	0.72 0.04
CH	0.81 0.03	0.91 0.01	0.71 0.05	0.96 0.00	0.81 0.03	0.79 0.11	0.91 0.01	0.96 0.01
ET	0.75 0.06	0.74 0.04	0.98 0.00	0.91 0.02	0.88 0.02	0.79 0.04	0.67 0.03	0.84 0.02
FI	0.39 0.07	0.29 0.06	0.52 0.04	0.68 0.06	0.89 0.03	0.68 0.15	0.74 0.04	0.89 0.02
IS	0.62 0.08	0.87 0.03	0.89 0.02	0.89 0.01	0.85 0.02	-	-	0.91 0.01
NO	0.70 0.02	0.42 0.04	0.59 0.04	0.69 0.02	0.70 0.02	0.65 0.18	0.76 0.02	0.88 0.02
NZ	0.66 0.04	0.85 0.03	0.67 0.06	0.85 0.02	0.91 0.02	0.02 0.07	0.82 0.03	0.88 0.02
SP	0.00 0.00	0.25 0.05	0.82 0.05	0.67 0.09	0.65 0.10	-	-	0.52 0.06
US	0.45 0.05	0.75 0.05	0.89 0.03	0.55 0.08	0.86 0.01	0.54 0.04	0.86 0.01	0.95 0.01
Mean	0.58	0.58	0.77	0.78	0.82	0.55	0.79	0.84
SD	0.26	0.31	0.15	0.14	0.09	0.27	0.08	0.14
CV	0.45	0.53	0.19	0.18	0.11	0.49	0.10	0.17

For each *Rhynchosporium* population (abbreviated by the country’s name) and trait, heritability (top line) and standard deviation (bottom line) are reported. The last three rows contain the mean, standard deviation (SD) and coefficient of variation (CV) across populations for each trait. For virulence, logit-transformed data was used to calculate heritability.

**Table 4:** Trait correlation matrix based on pooled isolates of the *Rhynchosporium commune* populations studied.

	Growth.rate 12°C	Growth rate18°C	Growthate 22°C	Fungicide resistance	Melanization	Sporesize	Sporenumber	Virulence
Growth rate 12°C								
Growth rate18°C	0.59^a^							
Growth rate 22°C	0.19	0.21^b^						
Fungicide resistance	−0.21^b^	−0.25^b^	−0.07					
Melanization	−0.25^b^	−0.39^a^	0.22^b^	0.07				
Spore size	−0.19	−0.25^b^	−0.13	0.02	−0.01			
Spore number	0.13	0.27^b^	0.05	−0.06	−0.03	−0.49^a^		
Virulence	0.08	0.12	0.11	0.01	0.22^b^	−0.16	0.21	

To make data comparable across populations, trait values of each trait-population combination were standardized to a mean of 0 and a standard deviation of 1, and then correlation analyses were performed based on isolate means. Sample sizes varied between 80 and 112.

a *P*<0.0018 (Bonferroni-corrected α for multiple comparisons = 0.05/28).

b *P*≤0.05.

## Discussion

The overall comparison of population divergence for neutral molecular markers and quantitative traits pointed toward a predominance of stabilizing selection in the barley pathogen *Rhynchosporium commune*. Five of eight traits revealed evidence for stabilizing selection across populations, indicating that the same phenotypes had been favoured across populations. These five traits were: growth rate at 18°C, melanization, spore size, spore number and virulence (Fig. 1). Three other traits did not follow this pattern. Growth rate at 12°C and fungicide resistance did not deviate from the neutral model of genetic drift as overall *Q*_ST_ was not significantly different from *F*_ST_. One trait, growth rate at 22°C, showed evidence for being under diversifying selection for local adaptation. The finding that growth at the stressful temperature of 22°C was under diversifying selection for local adaptation was consistent with the finding that mean growth was significantly correlated with mean annual temperature at the sites of origin [18]. A likely explanation for the general predominance of stabilizing selection among populations is that agricultural practices homogenize environmental variation, selecting for similar phenotypes around the world [7].

When our findings are compared to results from a meta-analysis including 62 studies on 50 species (54% plants, 24% vertebrates, 20% invertebrates, 2% fungi), stabilizing selection appears to be more common in *R. commune* than in many other species [4]. The reason is that 70% of the *Q*_ST_ values included in the meta-analysis were higher than the corresponding *F*_ST_ values, with the average difference being statistically significant [4]. Our results also differ in regard to the importance of genetic drift in natural populations. The meta-analysis on *Q*_ST_/*F*_ST_ comparisons revealed that the correlation of pairwise estimates of *Q*_ST_ and *F*_ST_ across studies was significant and positive [4], indicating the importance of genetic drift. In contrast, we found the two estimates to be poorly or negatively correlated for the eight traits measured, similar to findings of an older meta-analysis on *Q*_ST_/*F*_ST_ studies [3]. We therefore conclude that quantitative traits in *R. commune* are little affected by genetic drift, but they seem to be shaped by selection.

We are aware of only few studies where population differentiation for neutral markers and phenotypic traits were compared for fungi. A *Q*_ST_/*F*_ST_ comparison on similar traits in *Mycosphaerella graminicola* showed that for three traits (colony size, pycnidia size and percentage of leaf area covered with lesions) the effects of selection could not be separated from those of drift, while another three traits (fungicide resistance, temperature sensitivity and percentage leaf area covered with pycnidia) were under diversifying selection [26]. Only one trait, pycnidia density, was under stabilizing selection across populations. Evidence for stabilizing selection for virulence was found for *Melampsora lini* [30], but diversifying selection for virulence was found in *Puccinia striiformis* f. sp. *tritici* [31]. It therefore appears that our finding of the predominance of stabilizing selection has to be considered in a trait- and species-specific context. More studies will be needed to draw broad conclusions about the effects of anthropogenic homogenization of environmental conditions on the relative roles of genetic drift, stabilizing and diversifying selection.

Within-population genetic variation for expressed traits, calculated as heritability, was generally high (Table 3). Studies of agricultural pathogens often reveal very high heritabilities for traits thought to be closely associated with fitness. For example, heritable variation for growth varied between 0.5–0.8 in the wheat pathogen *Mycosphaerella graminicola* [32], [33] and between 0.8–0.9 for the fungal pathogen *Rhizoctonia solani* [34]. Analyses of virulence, where lesion area or length was measured in a controlled environment, reported heritabilities in the range of 0–0.6 for *Cochliobolus heterostrophus* [35], 0.87 for *Cochliobolus carbonum* [36] and 0.31–0.76 for *M. graminicola* [26]. For fungicide sensitivity, heritabilities for *in vitro* cyproconazole resistance were in the range of 0.17–0.80 in populations of *M. graminicola* [37]. For the pathogen *R. solani*, heritabilities for *in vitro* copper oxychloride sensitivity varied between 0.6–0.9 [34]. Compared to our study, mean heritabilities for spore output, measured as pycnidial density *in planta*, were only half as high in *M. graminicola* [26]. The relatively low heritability reported here for spore size is in agreement with findings from studies on other fungal species [26], [38].

The generally high heritability values are surprising because many of these traits are closely linked to a pathogen’s fitness, including growth rate under optimal temperature, spore number and virulence. A likely reason is that those traits have wide fitness functions [39]. Wide fitness functions could possibly be explained by some genetic correlations found among pairs of traits (Table 4). In contrast to the original interpretations of evolutionary theory of life histories [40], several strategies may exist within populations to achieve equal fitness. For example, our data set suggests that fungicide resistance tends to trade off against fast growth at optimal temperatures, while fast growth under such temperatures tends to trade off against spore size. We found evidence for a trade-off between the size and number of spores (offspring) produced, possibly associated with limited resources being available for reproduction. In addition, virulence tended to be positively correlated with spore number, as predicted by the trade-off theory of virulence evolution [19]. Also, our results did not support the often observed finding that life-history traits – thought to be under relatively strong directional selection, have lower heritabilities than morphological traits [41]. In our case heritability was lowest for spore size, intermediate for physiological traits (growth under different temperatures) and highest for life-history traits presumably strongly associated with fitness such as spore number and virulence.

In our study, average heritability across traits was significantly negatively correlated with gene diversity assessed for neutral microsatellite loci (Fig. 2). This contradicts the previous results of a meta-analysis that found no correlation between the two types of genetic variation [42]. It also contradicts quantitative genetics theory, which under many scenarios predicts either a positive relationship [43] or no relationship between the two, for example under somewhat large effective populations sizes combined with stabilizing selection [44] or under non-equilibrium situations in the presence of non-additive gene effects [45]. A reasonable hypothesis that could explain this unusual result is that the negative relationship is caused by an interaction between effective population size and the selection regime, where larger populations are more strongly exposed to variation-depleting selection. The relationship may be affected by the phylogeographic history of *R. commune*. The species emerged recently in Scandinavia through a host jump [46], and high neutral marker gene diversity may be maintained in Northern European populations through gene flow with wild relatives [47].

This study represents one of the first comparisons of the relative effects of genetic drift and selection on quantitative traits in an agriculturally important fungal pathogen. On the one hand, we found strong evidence that agricultural ecosystems impose similar selection on pest populations across large geographic regions, from Europe and North America to Africa and Australasia. On the other hand, these populations harbour very high levels of genetic variation for important pathogenicity traits, possibly due to wide fitness functions within populations. If this model is correct, it implies that similar selection combined with some gene flow creates a very large species-level population that is well-adapted to a wide variety of agricultural conditions. Therefore, species like *Rhynchosporium commune* harbour high evolutionary potential with the ability to adapt rapidly to simple disease management practices including the deployment of resistant cultivars and application of pesticides.

## Supporting Information

Table S1 **Pairwise** ***Q*** **_ST_ values for all eight quantitative traits studied and pairwise** ***F*** **_ST_ values for neutral SSR markers.**
(DOC)
