# Supplementary material for: The Influence of Genetic Drift and Selection on Quantitative Traits in a Plant Pathogenic Fungus
Source: PLoS One. 2014 Nov 10;9(11):e112523. doi: 10.1371/journal.pone.0112523 (PMC4226542; doi:10.1371/journal.pone.0112523)
Supplement: Table S1 — Pairwise Q ST values for all eight quantitative traits studied and pairwise F ST values for neutral SSR markers. (DOC) [file pone.0112523.s001.doc]

**Supporting information**

Pairwise *Q*_ST_ values for all eight quantitative traits studied and pairwise *F*_ST_ values for neutral SSR markers.

**Table S1**

A: *Q*_ST_ for growth rate at 12°C

|  | AU | CH | ET | FI | IS | NO | NZ | SP | US |  | Mean |
| --- | --- | --- | --- | --- | --- | --- | --- | --- | --- | --- | --- |
| AU | - |  |  |  |  |  |  |  |  |  | 0.25 |
| CH | 0.16 | - |  |  |  |  |  |  |  |  | 0.08 |
| ET | 0.08 | 0.00 | - |  |  |  |  |  |  |  | 0.09 |
| FI | 0.65 | 0.17 | 0.39 | - |  |  |  |  |  |  | 0.45 |
| IS | 0.00 | 0.12 | 0.03 | 0.65 | - |  |  |  |  |  | 0.22 |
| NO | 0.52 | 0.00 | 0.19 | 0.01 | 0.50 | - |  |  |  |  | 0.27 |
| NZ | 0.00 | 0.15 | 0.06 | 0.68 | 0.00 | 0.53 | - |  |  |  | 0.25 |
| SP | 0.25 | 0.00 | 0.00 | 0.62 | 0.18 | 0.22 | 0.28 | - |  |  | 0.19 |
| US | 0.33 | 0.00 | 0.00 | 0.45 | 0.26 | 0.15 | 0.32 | 0.00 | - |  | 0.19 |

B: *Q*_ST_ for growth rate at 18°C

|  | AU | CH | ET | FI | IS | NO | NZ | SP | US |  | Mean |
| --- | --- | --- | --- | --- | --- | --- | --- | --- | --- | --- | --- |
| AU | - |  |  |  |  |  |  |  |  |  | 0.11 |
| CH | 0.00 | - |  |  |  |  |  |  |  |  | 0.03 |
| ET | 0.00 | 0.00 | - |  |  |  |  |  |  |  | 0.04 |
| FI | 0.00 | 0.00 | 0.00 | - |  |  |  |  |  |  | 0.12 |
| IS | 0.04 | 0.01 | 0.00 | 0.17 | - |  |  |  |  |  | 0.12 |
| NO | 0.06 | 0.00 | 0.06 | 0.00 | 0.28 | - |  |  |  |  | 0.14 |
| NZ | 0.07 | 0.04 | 0.01 | 0.19 | 0.00 | 0.31 | - |  |  |  | 0.14 |
| SP | 0.60 | 0.16 | 0.27 | 0.40 | 0.47 | 0.05 | 0.47 | - |  |  | 0.38 |
| US | 0.09 | 0.01 | 0.00 | 0.22 | 0.00 | 0.36 | 0.00 | 0.60 | - |  | 0.16 |

C: *Q*_ST_ for growth rate at 22°C

|  | AU | CH | ET | FI | IS | NO | NZ | SP | US |  | Mean |
| --- | --- | --- | --- | --- | --- | --- | --- | --- | --- | --- | --- |
| AU | - |  |  |  |  |  |  |  |  |  | 0.26 |
| CH | 0.55 | - |  |  |  |  |  |  |  |  | 0.38 |
| ET | 0.36 | 0.71 | - |  |  |  |  |  |  |  | 0.48 |
| FI | 0.72 | 0.00 | 0.78 | - |  |  |  |  |  |  | 0.53 |
| IS | 0.00 | 0.47 | 0.33 | 0.63 | - |  |  |  |  |  | 0.22 |
| NO | 0.47 | 0.00 | 0.69 | 0.26 | 0.36 | - |  |  |  |  | 0.35 |
| NZ | 0.00 | 0.54 | 0.44 | 0.71 | 0.00 | 0.43 | - |  |  |  | 0.26 |
| SP | 0.00 | 0.39 | 0.23 | 0.60 | 0.00 | 0.31 | 0.00 | - |  |  | 0.19 |
| US | 0.00 | 0.37 | 0.26 | 0.52 | 0.00 | 0.26 | 0.00 | 0.00 | - |  | 0.18 |

D: *Q*_ST_ for fungicide resistance

|  | AU | CH | ET | FI | IS | NO | NZ | SP | US |  | Mean |
| --- | --- | --- | --- | --- | --- | --- | --- | --- | --- | --- | --- |
| AU | - |  |  |  |  |  |  |  |  |  | 0.08 |
| CH | 0.12 | - |  |  |  |  |  |  |  |  | 0.15 |
| ET | 0.00 | 0.30 | - |  |  |  |  |  |  |  | 0.20 |
| FI | 0.00 | 0.16 | 0.09 | - |  |  |  |  |  |  | 0.12 |
| IS | 0.01 | 0.32 | 0.00 | 0.10 | - |  |  |  |  |  | 0.20 |
| NO | 0.00 | 0.19 | 0.01 | 0.00 | 0.03 | - |  |  |  |  | 0.10 |
| NZ | 0.55 | 0.04 | 0.67 | 0.58 | 0.65 | 0.58 | - |  |  |  | 0.50 |
| SP | 0.00 | 0.00 | 0.22 | 0.00 | 0.20 | 0.00 | 0.38 | - |  |  | 0.10 |
| US | 0.00 | 0.10 | 0.32 | 0.00 | 0.29 | 0.00 | 0.56 | 0.00 | - |  | 0.16 |

E: *Q*_ST_ for melanization

|  | AU | CH | ET | FI | IS | NO | NZ | SP | US |  | Mean |
| --- | --- | --- | --- | --- | --- | --- | --- | --- | --- | --- | --- |
| AU | - |  |  |  |  |  |  |  |  |  | 0.00 |
| CH | 0.00 | - |  |  |  |  |  |  |  |  | 0.02 |
| ET | 0.00 | 0.00 | - |  |  |  |  |  |  |  | 0.02 |
| FI | 0.00 | 0.00 | 0.00 | - |  |  |  |  |  |  | 0.02 |
| IS | 0.00 | 0.04 | 0.04 | 0.00 | - |  |  |  |  |  | 0.04 |
| NO | 0.00 | 0.00 | 0.00 | 0.00 | 0.00 | - |  |  |  |  | 0.02 |
| NZ | 0.00 | 0.09 | 0.09 | 0.00 | 0.00 | 0.06 | - |  |  |  | 0.07 |
| SP | 0.00 | 0.03 | 0.00 | 0.15 | 0.21 | 0.10 | 0.23 | - |  |  | 0.09 |
| US | 0.00 | 0.00 | 0.00 | 0.01 | 0.06 | 0.00 | 0.11 | 0.00 | - |  | 0.02 |

F: *Q*_ST_ for spore size

|  | AU | CH | ET | FI | NO | NZ | US |  | Mean |
| --- | --- | --- | --- | --- | --- | --- | --- | --- | --- |
| AU | - |  |  |  |  |  |  |  | 0.09 |
| CH | 0.00 | - |  |  |  |  |  |  | 0.01 |
| ET | 0.00 | 0.00 | - |  |  |  |  |  | 0.00 |
| FI | 0.09 | 0.00 | 0.00 | - |  |  |  |  | 0.04 |
| NO | 0.00 | 0.00 | 0.00 | 0.00 | - |  |  |  | 0.02 |
| NZ | 0.48 | 0.00 | 0.00 | 0.00 | 0.06 | - |  |  | 0.13 |
| US | 0.00 | 0.09 | 0.02 | 0.15 | 0.08 | 0.24 | - |  | 0.10 |

G: *Q*_ST_ for spore number

|  | AU | CH | ET | FI | NO | NZ | US |  | Mean |
| --- | --- | --- | --- | --- | --- | --- | --- | --- | --- |
| AU | - |  |  |  |  |  |  |  | 0.11 |
| CH | 0.00 | - |  |  |  |  |  |  | 0.07 |
| ET | 0.27 | 0.18 | - |  |  |  |  |  | 0.11 |
| FI | 0.00 | 0.00 | 0.13 | - |  |  |  |  | 0.05 |
| NO | 0.02 | 0.00 | 0.06 | 0.00 | - |  |  |  | 0.03 |
| NZ | 0.04 | 0.01 | 0.00 | 0.00 | 0.00 | - |  |  | 0.02 |
| US | 0.30 | 0.22 | 0.00 | 0.17 | 0.11 | 0.05 | - |  | 0.14 |

H: *Q*_ST_ for virulence

|  | AU | CH | ET | FI | IS | NO | NZ | SP | US |  | Mean |
| --- | --- | --- | --- | --- | --- | --- | --- | --- | --- | --- | --- |
| AU | - |  |  |  |  |  |  |  |  |  | 0.20 |
| CH | 0.15 | - |  |  |  |  |  |  |  |  | 0.03 |
| ET | 0.08 | 0.00 | - |  |  |  |  |  |  |  | 0.04 |
| FI | 0.24 | 0.00 | 0.00 | - |  |  |  |  |  |  | 0.05 |
| IS | 0.00 | 0.00 | 0.00 | 0.04 | - |  |  |  |  |  | 0.07 |
| NO | 0.23 | 0.00 | 0.00 | 0.00 | 0.04 | - |  |  |  |  | 0.05 |
| NZ | 0.50 | 0.07 | 0.25 | 0.13 | 0.34 | 0.12 | - |  |  |  | 0.21 |
| SP | 0.05 | 0.00 | 0.00 | 0.00 | 0.00 | 0.00 | 0.30 | - |  |  | 0.05 |
| US | 0.31 | 0.00 | 0.03 | 0.00 | 0.13 | 0.00 | 0.00 | 0.08 | - |  | 0.07 |

I: *F*_ST_ based on 8 neutral SSR markers

|  | AU | CH | ET | FI | IS | NO | NZ | SP | US |  | Mean |
| --- | --- | --- | --- | --- | --- | --- | --- | --- | --- | --- | --- |
| AU | - |  |  |  |  |  |  |  |  |  | 0.19 |
| CH | 0.29 | - |  |  |  |  |  |  |  |  | 0.30 |
| ET | 0.29 | 0.42 | - |  |  |  |  |  |  |  | 0.30 |
| FI | 0.10 | 0.26 | 0.23 | - |  |  |  |  |  |  | 0.13 |
| IS | 0.20 | 0.35 | 0.35 | 0.11 | - |  |  |  |  |  | 0.21 |
| NO | 0.14 | 0.26 | 0.25 | 0.00 | 0.07 | - |  |  |  |  | 0.13 |
| NZ | 0.12 | 0.26 | 0.26 | 0.09 | 0.24 | 0.14 | - |  |  |  | 0.18 |
| SP | 0.16 | 0.29 | 0.24 | 0.06 | 0.17 | 0.07 | 0.08 | - |  |  | 0.16 |
| US | 0.22 | 0.30 | 0.34 | 0.16 | 0.18 | 0.13 | 0.23 | 0.21 | - |  | 0.22 |
